# The Evolutionary History of the Extinct Baltic Sea Harp Seal Population

**DOI:** 10.1002/ece3.71322

**Published:** 2025-05-08

**Authors:** Maiken Hemme Bro‐Jørgensen, Hans Ahlgren, Aikaterini Glykou, Emily J. Ruiz‐Puerta, Lembi Lõugas, Anne Birgitte Gotfredsen, Morten Tange Olsen, Kerstin Lidén

**Affiliations:** ^1^ Archaeological Research Laboratory, Department of Archaeology and Classical Studies Stockholm University Stockholm Sweden; ^2^ Section for Molecular Ecology and Evolution, Globe Institute University of Copenhagen Copenhagen Denmark; ^3^ Arctic Centre/Groningen Institute of Archaeology, Faculty of Arts University of Groningen Groningen the Netherlands; ^4^ Archaeological Research Collection Tallinn University Tallinn Estonia; ^5^ Section for GeoGenetics, Globe Institute University of Copenhagen Copenhagen Denmark

**Keywords:** ancient DNA, Baltic Sea, breeding population, environmental change, harp seal, mitogenome

## Abstract

The now‐extinct harp seal population that inhabited the Baltic Sea from the Mesolithic to the Iron Age is an enigma. It occurred outside the species' contemporary Arctic range, likely deviated from typical harp seal migratory behaviour, and experienced body size reductions and dramatic population fluctuations leading up to its extinction. Here we use ancient DNA analyses to shed more light on the evolutionary history of the Baltic Sea harp seal population, including its origin, timing of colonisation, diversity and factors contributing to its demise. We generated 49 ancient Baltic and eight ancient Arctic harp seal mitogenomes, which we analysed together with 53 contemporary Arctic harp seal mitogenomes. We detected limited phylogeographic resolution among ancient and contemporary populations, which we interpret as a late Pleistocene range expansion from a common refugial population with subsequent gene flow. Ancient Baltic harp seals were significantly genetically differentiated from contemporary harp seal populations and retained their own genetic composition throughout time. The genetic diversity of Baltic harp seals decreased over time, yet was comparable to that of contemporary populations. This suggests that Baltic harp seals formed a distinct breeding population, which may occasionally have received immigrants from the Arctic but was itself confined in the Baltic Sea until the end. We hypothesise that loss of genetic diversity and the ultimate extinction of the Baltic harp seal population was a consequence of population fluctuations caused by climatic change, reduced salinity and biological productivity, and periodic intense human harvest.

## Graphical abstract

The figure exhibits the spatiotemporal distribution of harp seal mitogenome clades, showing temporal changes in ancient Baltic Sea populations and spatial differences among modern populations from Newfoundland and the White Sea. Key localities analysed include ancient and modern samples from the Baltic Sea, Finnmark, White Sea, Greenland Sea, and Newfoundland, highlighting variations in mitogenome clades over time and across regions.

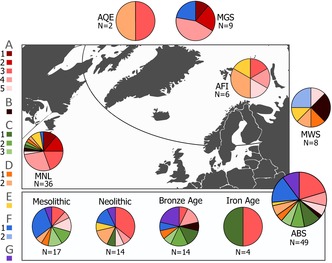

## 1 Introduction

It is increasingly clear that human activities have had a significant and often underestimated impact on marine ecosystems, either being the main driver of population declines and extinctions or acting in concert with environmental change (Ahlgren et al. 2022; Keighley et al. 2019). The semi‐enclosed brackish water of the Baltic Sea is, arguably, one of the most severely impacted aquatic environments on Earth (Dietz et al. 2021). It was formed as a freshwater lake following the last glacial retreat and attained its current form ~8000 BP. During its relatively short history, it has experienced multiple rapid ecological shifts from freshwater to marine environments (Emeis et al. 2003), as well as a long list of human impacts, from late Pleistocene seal hunting (Ahlgren et al. 2022) to 21st century pollution (Sonne et al. 2020). Five marine mammal species have been present in the Holocene Baltic Sea: the grey seal ( *Halichoerus grypus* ), harbour seal ( *Phoca vitulina* ), ringed seal ( *Pusa hispida* ), harp seal ( *Pagophilus groenlandicus* ), and the harbour porpoise *Phocoena phocoena* ) (Schmölcke 2008; Sommer et al. 2008; Storå and Lõugas 2005). These species are all believed to have colonised the region shortly after the last glacial retreat and gradually moved into the newly formed Baltic Sea as this became available. The subfossil record suggests that they subsequently followed very contrasting demographic trajectories, with grey and ringed seals typically being widespread and abundant, although with brief periods of lower abundance; harbour seals and porpoises persisting at low abundance; and harp seals becoming locally extinct (Ahlgren et al. 2022; Olsen et al. 2018; Sommer et al. 2008; Ukkonen 2002; Ukkonen et al. 2014).

Radiocarbon dating and zooarchaeological analysis of harp seal remains from numerous archaeological sites in the Baltic Sea region suggest two phases of presence in the Baltic Sea (Glykou et al. 2021). The harp seal is the dominant seal species in faunal assemblages during the first phase (c. 6500–4500 BP), which intriguingly is characterised by a gradual reduction in body size (Storå and Ericson 2004). This phase is followed by a hiatus of almost 1000 years with no harp seal finds until they reappear in a second phase starting around the early Bronze Age (c. 3700–3300 BP). During this second phase, harp seal subfossils are represented by significantly lower relative frequencies in the faunal remains compared to the first phase and eventually, in the late Iron Age–Medieval period (Aguraiuja‐Lätti et al. 2022; Lõugas and Aguraiuja‐Lätti 2023) the harp seal disappears from the Baltic Sea zooarchaeological record. Morphometric analysis of harp seal bones has led to the identification of pups (0–3 months old) at sites dated to the Late Mesolithic and Early Neolithic (c. 6400–5800 BP), documenting the existence of a breeding harp seal population in the southwestern Baltic Sea during the Late Atlantic climatic period (Glykou 2014; Glykou et al. 2021). Likewise, the identification of pups in the faunal assemblages of the Middle–Late Neolithic (c. 5000–4400 BP) indicates the presence of another breeding harp seal colony in the Baltic Proper, most likely between the Åland islands and the island of Gotland (Storå 2001; Storå and Ericson 2004).

The past, long‐term occurrence of breeding harp seal colonies in the Baltic Sea is intriguing considering the species' biology, life‐history and current Arctic distribution (Storå and Ericson 2004). Contemporary harp seals rely on the presence of stable pack ice as a breeding, resting and moulting habitat, with newborn pups tied to the pack ice until they are weaned and have attained their adult fur a few weeks after birth (Johnston et al. 2012; Ronald and Dougan 1982; Stenson and Hammill 2014). During this period, harp seals aggregate in huge numbers on the pack ice in the White Sea, the Greenland Sea and off Newfoundland, and for the rest of the year the species undertake long‐distance foraging migrations across much of the Arctic and Subarctic North Atlantic, including occasional visits to the North Sea (Nilssen et al. 1998; Sergeant 1991).

Here, we analyse 110 ancient and modern harp seal mitogenomes to shed additional light on the natural history of the extirpated Baltic Sea harp seal population. First, we use ancient DNA analysis to obtain 49 ancient mitogenomes from harp seal subfossils from Mesolithic, Neolithic, Bronze and Iron Age archaeological sites in the Baltic Sea region, as well as eight ancient mitogenomes from sites in western Greenland and northern Norway. Next, we perform Bayesian phylogenetic analyses, construct haplotype networks and estimate genetic diversity and differentiation for the ancient mitogenome data together with 53 previously published modern mitogenomes representing the species' contemporary breeding range (Carr et al. 2015). Our aim is to address multiple unanswered questions regarding the now‐extinct Baltic harp seal population. Specifically, we aim to determine whether it formed a genetically distinct population, to identify its origin, assess the possibility of multiple colonisation events in the Baltic, and examine if there are signs of a loss of genetic diversity preceding its extirpation during the late Iron Age–Medieval period.

## 2 Materials and Methods

### 2.1 DNA Extraction and Sequencing

**FIGURE 1:**
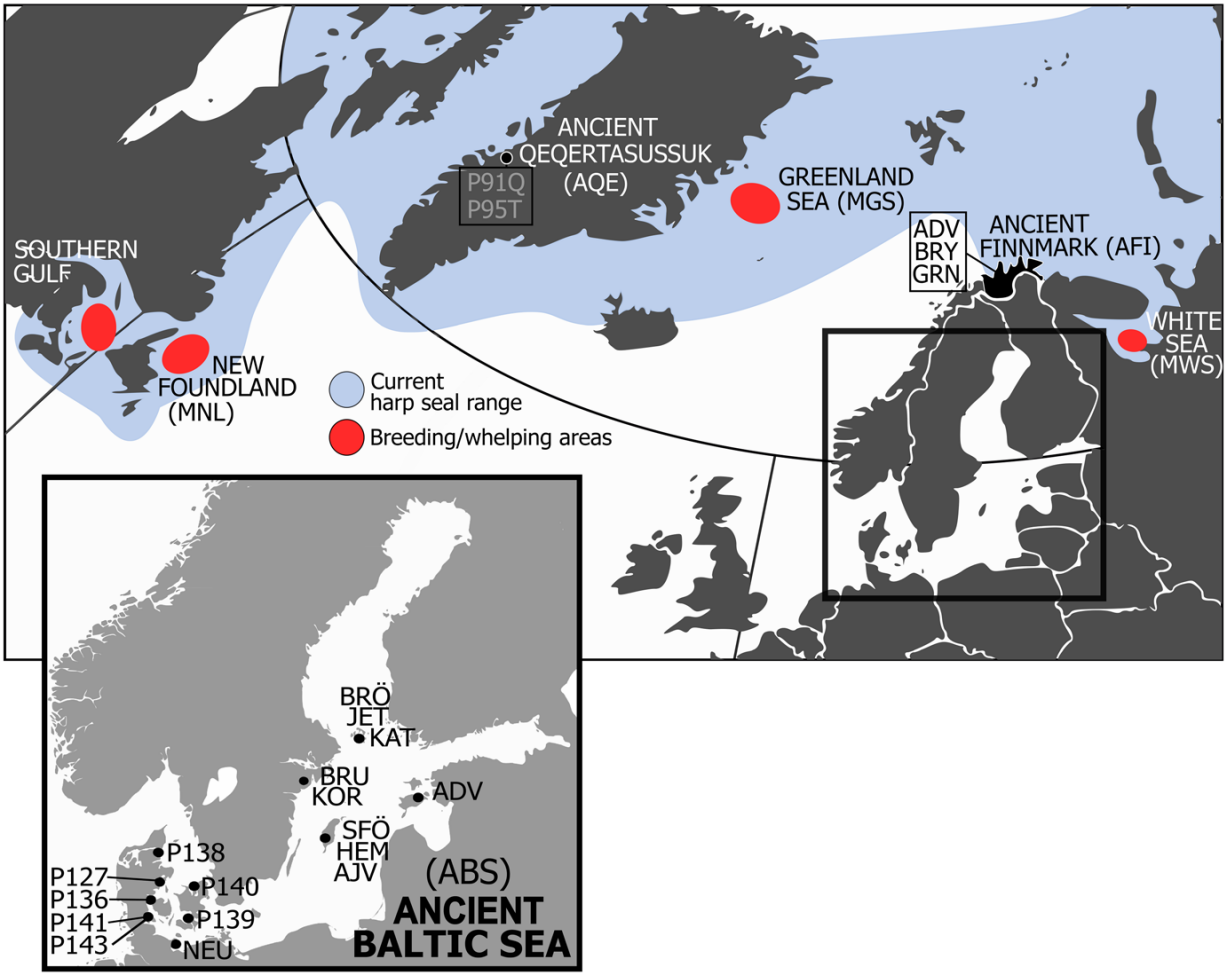
Harp seal sample localities. Top: Map of harp seal's current distribution and breeding/whelping sites, including sampling localities from contemporary harp seals (red; Carr et al. 2015) and our ancient DNA sampling localities. Localities include: ABS, ancient Baltic Sea; AFI, ancient Finnmark; AQE, ancient Qeqertasussuk; MGS, modern Greenland Sea; MNL, modern Newfoundland; MWS, modern White Sea. Down left: Detailed map of sampling localities used for ancient DNA analyses of the now‐extinct Baltic Sea harp seal.

Ancient DNA was extracted from harp seal bone samples from archaeological sites in the Baltic Sea region (Denmark, Estonia, Germany, Sweden and Finland), Qeqertasussuk in the Northwest Atlantic (Disko Bay in West Greenland) and in eastern Finnmark (Norway) (Figure 1; Table S1). The Danish samples were previously radiocarbon dated by Bennike et al. (2008), as were some of the samples from Germany, Sweden, Finland and Estonia (Glykou et al. 2021). The samples from Finnmark were indirectly dated from the archaeological contexts in which they were found. The samples from Qeqertasussuk were identified as belonging to the Saqqaq culture (Grønnow 2017). Both the Finnmark and the Qeqertasussuk samples correspond in age to the Neolithic Period in the Baltic Sea.

All bone material was drilled with a dentist drill to obtain bone powder, from which DNA was extracted using two different strategies; In the ancient DNA laboratory at the Archaeological Research Laboratory, Stockholm University, Sweden, bone powder was digested with a lysis buffer consisting of EDTA (0.5 M, pH 8), Triton X and Proteinase K (100 mg/mL), using a modified version of protocol C by Yang et al. (1998) followed by extract concentration using Amicon Ultra Centrifugal Filters (Sigma‐Aldrich, Darmstadt, Germany) and elution using MinElute‐spin columns (Qiagen, Hilden, Germany). In the ancient DNA laboratory at University of Copenhagen's Globe Institute, Denmark, digestion was performed with a lysis buffer consisting of EDTA (0.5 M, pH 8), Urea (1 M) and Proteinase K (10 mg/μL), followed by elution using Zymo‐spin reservoirs (Zymo Research, CA, USA) in combination with MinElute‐spin columns (Qiagen, Hilden, Germany) (Dabney et al. 2013). All ancient DNA libraries were built following the protocol of Meyer and Kircher (2010) and sequenced at SciLifeLab Stockholm, Sweden, using the Illumina HiSeq X (PE 150 bp), Illumina HiSeq2500 (PE 125 bp), and Illumina NovaSeq S1 (PE 150 bp) sequencing platforms.

### 2.2 DNA Data Quality Control and Alignment

Initial processing and authentication of the ancient DNA sequence data were carried out through the PALEOMIX pipeline (v.1.2.13.1) (Schubert et al. 2014), as described in detail in Supporting Information. Briefly, we used AdapterRemoval (v.2.2.0) (Schubert et al. 2016) to remove adapter DNA from the sequences, Picard MarkDuplicates to remove PCR duplicates, BWA to align sequences to a harp seal mitogenome (NCBI GenBank accession KP942581) and GATK Indel realigner to realign BAM files, sorting of which were enabled with SamTools (v.0.1.19) (Li et al. 2009). MapDamage (v.2.0.9) (Ginolhac et al. 2011) was used to assess the post mortem damage, confirming the authenticity of the aDNA. Realigned bam files were exported to the program Geneious Prime v. 2020.1.2 (https://www.geneious.com) and re‐mapped (NCBI GenBank accession KP942581) in order to generate 57 ancient near‐complete (15,825 bp) mitogenomes, excluding the control region. Upon visual inspection, three sample pairs NEU17‐BRU44, BRY3‐BRY5, and NEU7‐NEU14, respectively, were identified as having identical mitogenome sequences. The samples NEU17 and BRU44 are from two different localities and therefore must represent two different individuals. Although NEU7 and NEU14 are from the same locality and time period, they have different sex (Bro‐Jørgensen et al. 2021) and therefore must be different individuals. The samples BRY3 and BRY5 are from the same locality and time period; they are both males, represented by femurs; however, their morphology indicates that they originate from two different individuals. The sample pairs were therefore treated as separate individuals in all analyses. Finally, we compared our ancient data with 53 previously published mitogenomes from contemporary harp seal breeding populations in Newfoundland (the Labrador ice front together with the Gulf of Saint Lawrence), the Greenland Sea and the White Sea (Carr et al. 2015).

### 2.3 Phylogeny and Haplotype Network

The phylogenetic relationship of Baltic Sea harp seals and their temporal origin was assessed by tip‐age calibrated phylogenetic analysis of 110 samples (57 ancient, 53 modern) in BEAST 2 (v.2.5.1) (Bouckaert et al. 2014, 2019). First, we used bModelTest (Bouckaert and Drummond 2017) to determine the appropriate substitution model, gamma rate heterogeneity, and proportion of invariable sites for the mitogenome sets. Next, tree and clock models and sample tip ages were defined in the graphical user‐interface BEAuTI2 (v.2.6.3). We used uncalibrated data points for those samples that had radiocarbon dating results, while the remaining samples were assigned rough time points based on their approximate dating (Table S1). Specifically, following the recommendation from bModelTest (Bouckaert and Drummond 2017), we used a modified GTR substitution model, a Coalescent exponential population model, and a relaxed clock exponential model (Drummond et al. 2006). Analyses were conducted using a Markov chain Monte Carlo (MCMC) model consisting of 300 million iterations, with a burn‐in of 10%, and sampling of every 3000 trees. Runs were checked for convergence in Tracer (v.1.7.1) to ensure minimum Estimated Sample Size (ESS) values of ≥ 200 (Rambaut et al. 2018). Output tree files from each run were combined in LogCombiner (v.2.6.3) and analysed in TreeAnnotator (v.2.6.0) with a maximum clade credibility tree as the target tree type. In addition to the phylogenetic analyses, the relationship among ancient and modern harp seal mitogenomes was assessed by constructing a median‐joining haplotype network in PopART v. 1.7 (Bandelt et al. 1999; Leigh and Bryant 2015).

### 2.4 Genetic Diversity and Differentiation

The program DnaSP v.6 (Rozas et al. 2003) was used to estimate the haplotype (*H*_d_) and nucleotide diversity (*P*_i_) per locality and time period, as well as *K*_ST_ and *K*_ST_* values as a measure of the level of pairwise genetic differentiation between the sample groups defined by locality and time period.

## 3 Results

### 3.1 Phylogeny and Haplotype Network

**FIGURE 2:**
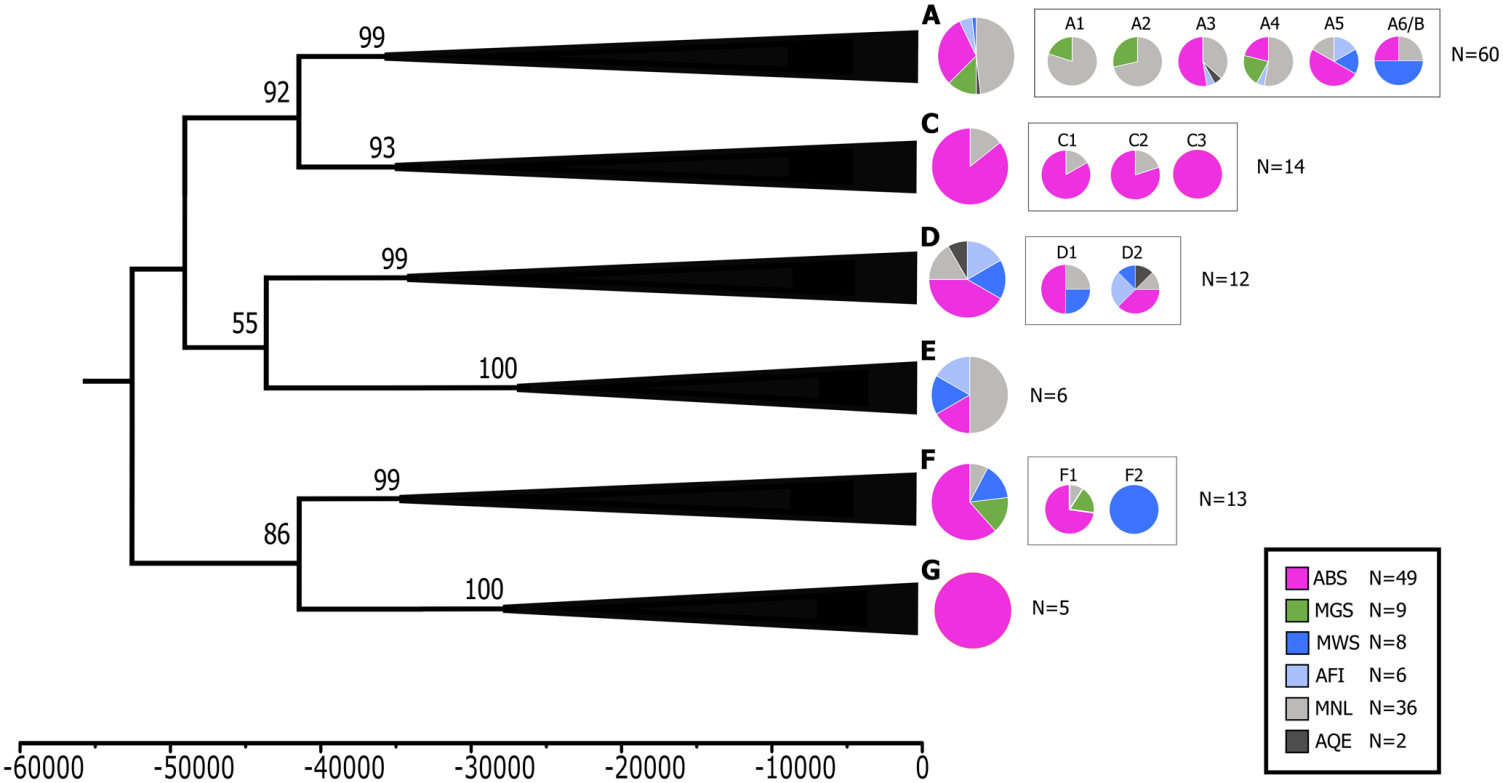
Simplified representation of the time‐dated Bayesian phylogeny with a pie chart showing the proportion of samples from each locality within each of the seven evolutionary clades, as well as support values for clades and basal branches. Localities include: ABS, ancient Baltic Sea; AFI, ancient Finnmark; AQE, ancient Qeqertasussuk; MGS, modern Greenland Sea; MNL, modern Newfoundland; MWS, modern White Sea. Subclade representation is shown as smaller pie charts in boxes next to the pie chart representing the entire clade. Time is indicated in years before present. The full phylogeny is provided in Figure S1.

**FIGURE 3:**
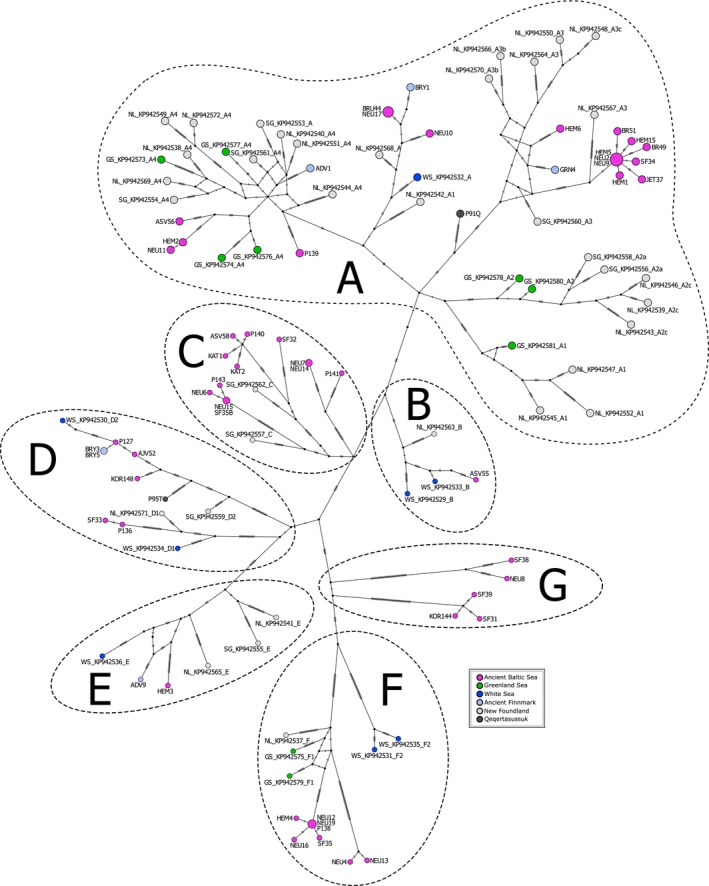
Median‐joining haplotype network of ancient and modern harp seals with stippled circles outlining the seven main evolutionary clades A–G. For ancient samples, see Table S1. Modern samples from Carr et al. (2015).

The tip‐dated Bayesian phylogeny and haplotype network point to the existence of seven main harp seal mitogenome clades A–G (Figures 2 and 3; Figure S1). Clades A–F correspond to the haplogroups identified by Carr et al. (2015), while we suggest the classification of a new clade G. In the phylogeny, all clades show high posterior support values (0.93–1.00); however, the basal topology denoting the relationship among clades is associated with some uncertainty with lower posterior support values (0.55–0.92). All seven major clades appear to share a common ancestor about 55,000 BP, followed by a split into different clades of around 50,000–35,000 BP. The subclades within clades D and F appear to arise about 35,000 BP, whereas subclades within C and the large clade A arise 35,000–20,000 BP. Finally, we see a diversification of haplotypes (coalescence events) across the phylogenetic tree about 15,000–8000 BP.

**FIGURE 4:**
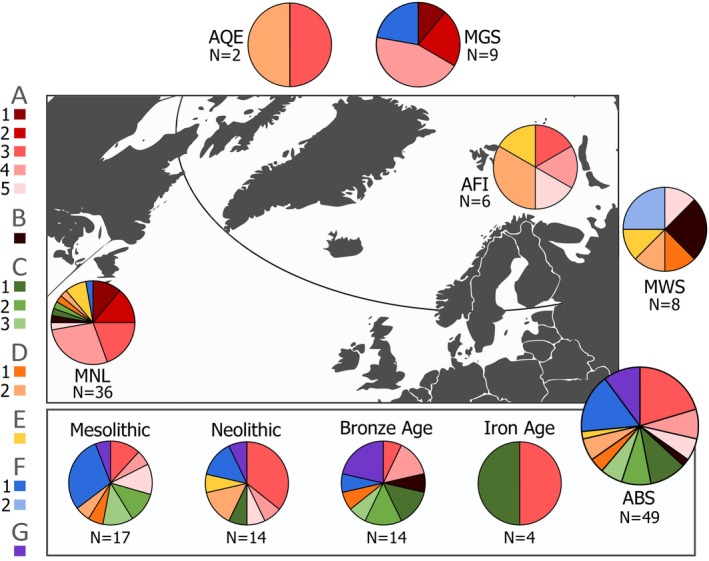
Spatiotemporal distribution of the main harp seal mitogenome clades (A–G) and their subclades. The analyses indicate some temporal changes in ancient Baltic Sea haplotype frequencies, as well as spatial differences among modern samples from Newfoundland and White Sea. Localities include: ABS, ancient Baltic Sea; AFI, ancient Finnmark; AQE, ancient Qeqertasussuk; MGS, modern Greenland Sea; MNL, modern Newfoundland; MWS, modern White Sea.

The phylogenetic tree and haplotype network are characterised by a limited spatiotemporal structure, with samples from distinct localities and time‐periods spread across clades (Figures 2 and 3; Figure S1). This also applies to the ancient Baltic Sea harp seal samples, which are distributed among all seven main mitogenome clades. Notably, ancient Baltic Sea samples tend to occur at higher frequencies within clades C, F, and G, accounting for approximately 86%, 62%, and 100% of the samples from all sites, respectively (Figure 3). In particular, we note that clade G is exclusively found in the ancient Baltic Sea, although it cannot have originated in the Baltic Sea, and clade C is almost exclusively found in the Baltic, with only two out of 14 haplotypes in this clade occurring outside the Baltic. In terms of temporal patterns, the majority of Baltic Sea harp seal mitogenome clades are represented already in the Mesolithic period, as demonstrated by samples from the western Baltic Sea (i.e., Neustadt, Flynderhage and Ronæs Skov), and these clades continue to be present throughout the Neolithic and the Bronze Age (Figure 4). In the Iron Age, only clade A and C haplotypes are observed in Baltic Sea harp seals; however, this may be due to a small sample size.

### 3.2 Genetic Diversity and Differentiation

In our estimates of spatiotemporal genetic differentiation, both the *K*_ST_ and *K*_ST_* values suggest that the ancient Baltic harp seal populations are genetically differentiated from the modern harp seal populations (Table 1). The ancient Baltic Sea harp seals have the highest differentiation to modern harp seals from Newfoundland (*K*_ST_ = 0.045 and *K*_ST_* = 0.019), intermediate to Greenland Sea (*K*_ST_ = 0.018 and *K*_ST_* = 0.010) and lowest to White Sea (*K*_ST_ = 0.014 and *K*_ST_* = 0.007) harp seals. The modern harp seal populations are all significantly differentiated from each other, except for the Greenland Sea and Newfoundland harp seals. In contrast, the genetic differentiation among ancient harp seals from the Baltic Sea, West Greenland and Finnmark is low and non‐significant.

**TABLE 1:** Genetic differentiation of harp seal localities and time periods estimated as pairwise *K*_ST_ and *K*_ST_* calculated in DnaSP v. 6.

	ABS	ABS Mesolithic	ABS Neolithic	ABS Bronze Age	ABS Iron Age	AFI	MWS	AQE	MGS	MNL
ABS		—	—	—	—	0.007	0.014*	−0.005	0.018*	0.045***
ABS Mesolithic	—		−0.006	−0.003	0.040*	0.016	0.024	−0.009	0.031	0.053***
ABS Neolithic	—	−0.006		0.002	0.011	−0.004	0.024	−0.023	0.026	0.030**
ABS Bronze Age	—	−0.004	0.001		0.028	0.024	0.022	−0.013	0.043*	0.045***
ABS Iron Age	—	0.008	−0.008	0.001		0.090	0.097*	0.020	0.098**	0.030**
AFI	0.005	0.008	0.008	0.014	0.050		0.014	−0.079	0.049	0.021*
MWS	0.007*	0.012	0.016	0.008	0.031*	0.010		−0.026	0.053*	0.033**
AQE	0.003	0.005	0.006	0.005	0.103*	0.032	0.011		−0.004	−0.004
MGS	0.010*	0.017*	0.021*	0.017*	0.044**	0.031*	0.023*	0.040		0.007
MNL	0.019***	0.020***	0.017***	0.015***	0.010**	0.010**	0.010**	0.007	0.002	

*Note:* *K*_ST_ is shown above the diagonal and *K*_ST_* is shown below the diagonal. Numbers give the estimated degree of genetic differentiation. Significant values are marked in bold and the level of significance is denoted by the number of asterisks, *0.01 < *p* < 0.05; **0.001 < *p* < 0.01; ***p < 0.001.

Abbreviations: Localities include: ABS, Ancient Baltic Sea; AFI, ancient Finnmark; AQE, ancient Qeqertasussuk; MGS, modern Greenland Sea; MNL, modern Newfoundland; MWS, modern White Sea.

In the Baltic Sea, the level of genetic differentiation among time periods is close to zero and non‐significant, with the exception of some differentiation between Mesolithic and Iron Age harp seals (*K*_ST_ = 0.040 and *K*_ST_* = 0.008). Similarly, there were relatively low and non‐significant levels of genetic differentiation between ancient Finnmark and modern White Sea harp seals, as well as between ancient Qeqertasussuk in West Greenland and modern Newfoundland–Greenland Sea harp seals. However, the sample sizes from ancient Finnmark and Qeqertasussuk, as well as Baltic Iron Age, are small and the results should be considered with caution.

**FIGURE 5:**
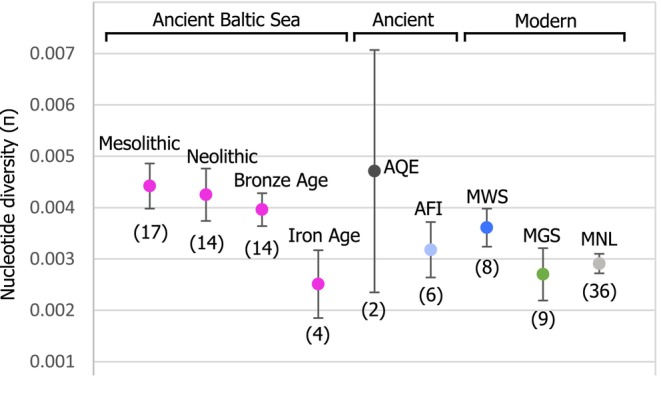
The mitogenome nucleotide diversity was substantially higher in Mesolithic and Neolithic harp seals from the Baltic Sea compared to contemporary populations, but declined towards the extirpation of harp seals in the Baltic Sea. The error bars show the standard variation (SD), and the number of samples is given below each data point. The exact values are found in Table 1. ABS, Ancient Baltic Sea; AFI, ancient Finnmark; AQE, ancient Qeqertasussuk; MGS, modern Greenland Sea; MNL, modern Newfoundland; MWS, modern White Sea.

The haplotype diversity was overall high at all localities and time periods, with most individuals carrying unique haplotypes, besides a few individuals from ancient Finnmark and Mesolithic Baltic Sea (Table 2). In terms of nucleotide diversity, all modern populations of harp seals have significantly lower values compared to the now‐extinct Baltic Sea harp seals, in particular from the Mesolithic and Neolithic periods (Table 2; Figure 5). Interestingly, Baltic Sea harp seals show a trend of decreasing nucleotide diversity with time from the Mesolithic towards the Iron Age, after which they disappeared from the Baltic Sea zooarchaeological record.

**TABLE 2:** DnaSP 6 measures of DNA polymorphism: Number of sequences, number of polymorphic sites (*S*), number of haplotypes (*h*), haplotype (gene) diversity (*H*_d_), nucleotide diversity (*P*_i_) and the standard deviation (SD) of the nucleotide diversity.

Global	Number of sequences	Number of polymorphic sites (*S*)	Number of haplotypes (*h*)	Haplotype (gene) diversity (*H*_d_)	Nucleotide diversity (*P*_i_)	Nucleotide diversity standard deviation (SD)
MNL	36	437	36	1.000	0.00291	0.00019
MGS	9	130	9	1.000	0.00270	0.00051
MWS	8	175	8	1.000	0.00361	0.00037
AFI	6	113	5	0.9333	0.00316	0.00042
AQE	2	74	2	1.000	0.00471	0.00236
ABS	49	506	44	0.994	0.00412	0.00024
Mesolithic	17	337	15	0.985	0.00442	0.00044
Neolithic	14	303	14	1.000	0.00425	0.00051
Bronze Age	14	294	14	1.000	0.00396	0.00032
Iron Age	4	63	4	1.000	0.00251	0.00066

Abbreviations: ABS, Ancient Baltic Sea; AFI, ancient Finnmark; AQE, ancient Qeqertasussuk; MGS, modern Greenland Sea; MNL, modern Newfoundland; MWS, modern White Sea.

## 4 Discussion

### 4.1 A Distinct Baltic Harp Seal Breeding Population

The mitogenome phylogenetic analyses reveal that the extirpated Baltic harp seals did not represent their own evolutionary clade, agreeing with contemporary harp seals, which show limited phylogeographic structure (Carr et al. 2015). Still, we note that mitogenome clade C is mainly and clade G exclusively found in Baltic harp seals. Furthermore, estimates of pairwise genetic differentiation clearly separate this population from contemporary harp seal populations. Thus, taken together with zooarchaeological finds of harp seal pups across much of the Baltic Sea region (Glykou 2014, 2016; Glykou et al. 2021), all available evidence points to the existence of a distinct harp seal breeding population in the Baltic Sea in the past.

The origin of this Baltic Sea harp seal breeding population has been much debated (Bennike et al. 2008; Storå and Ericson 2004), with recent studies suggesting that the White Sea is the most likely source of colonisers (Glykou et al. 2021). Our mitogenome data provide a slightly more nuanced explanation. The phylogenetic tree and haplotype network show clear separation into multiple distinct evolutionary clades, although these lack a clear geographical pattern. We interpret this as all analysed ancient and contemporary harp seal breeding populations—Baltic, White Sea, Greenland Sea, and Labrador—originated, expanded, and diversified from a common source, followed by alternating periods of semi‐isolation and admixture. The dated Bayesian phylogenetic analyses suggest that the observed haplotypes all originate from a single, relatively young evolutionary lineage prior to 55,000 BP, after which they started to diversify and over a short time span gave rise to the major observed clades by 35,000 BP. The existence of a single harp seal haplotype lineage coincides with the end of the Middle Weichselian Glacial Maximum (MWGM; MIS 4; 71,000–57,000 BP); a period characterised by rapid global cooling, build‐up of polar ice sheets, and changes in deep ocean circulation (Shackleton et al. 2021). Recent studies suggest that the MWGM was equally cold as the last glacial maximum (LGM; MIS 2; 29,000–14,000 BP), but characterised by more extensive glaciations (Doughty et al. 2021; Seidenkrantz et al. 2019), likely limiting the extent of suitable harp seal habitats and causing severe population declines. The diversification of haplotypes occurring 55,000–35,000 BP coincides with a period of long climate interstadials (warm periods) during MIS 3 (59,000–31,000 BP), which was sufficient to efficiently reduce the size of the Fennoscandian Ice Sheet (Kleman et al. 2021), and hence increase the amount of harp seal habitat in the North Atlantic. We suggest that this led to an increase in harp seal population size and haplotype diversity, perhaps coupled with some isolation and diversification of major lineages (clades) in large pockets of available habitat.

The current haplotype distribution points to substantial admixture sometime after the initial diversification of the major clades, perhaps during the LGM, when the size of the harp seal habitat once again declined. We note that certain clades appear to have remained more associated with certain geographical regions, such as the case with clades C and G mainly or exclusively found in the now‐extinct Baltic Sea harp seals, as well as clade B in White Sea harp seals, which could point to the existence of multiple LGM refugia.

### 4.2 A Single Baltic Sea Colonisation Event Followed by Periodic Immigration

The initial colonisation of the Baltic Sea by harp seals is assumed to have occurred during the Holocene, with the first zooarchaeological records dating back to approximately 7000 BP (Bennike et al. 2008; Ukkonen 2002). Further, osteoarchaeological evidence suggests the presence of an established breeding population in the southwest Baltic Sea in the Late Mesolithic by 6400–5800 BP (Glykou 2016), and in the Baltic proper in the Middle Mesolithic (Storå and Ericson 2004). Older findings dating back to 13,000–11,000 BP have been reported in the neighbouring Skagerrak‐Kattegat, suggesting the presence of harp seals in the region during the Late Pleistocene–Early Holocene (Aaris‐Sørensen 2010). Our phylogenetic analyses support these findings, with most of the ancient and modern mitogenome haplotypes arising after the LGM, including the clades C and G that primarily occurred in the Baltic Sea. In contrast, Baltic Sea ringed seals appear to have a more complex colonisation history, likely including multiple colonisation events and glacial refugia, some predating the LGM (Olsen et al. 2025; Sromek et al. 2024).

Considering the dynamics and extent of the LGM ice sheet (Hughes et al. 2016) we interpret the genetic and zooarchaeological data as a stepwise colonisation of the Baltic Sea, initiating in a marine paleo‐Skagerrak–Kattegat area, tracking the receding ice southward to the southwestern Baltic and ultimately moving up into the Baltic proper. Our genetic data indicate that connection with other breeding populations was likely reduced, as we observe increasing levels of genetic differentiation from other populations throughout the Baltic harp seal presence, from the Mesolithic to the Iron Age. In support of this, strontium isotope (^87^Sr/^86^Sr) analyses of 11 individuals indicate that harp seals rarely migrated out of the Baltic Sea, except for one animal, which appeared to have spent some time during gestation in the Atlantic Ocean and then entered the Baltic Sea to give birth (Glykou et al. 2018, 2021).

Upon establishment of a breeding population, harp seals appear omnipresent in the Baltic Sea until the Late Neolithic, when the zooarchaeological record is marked by a striking 1000‐year hiatus. Harp seals then reappear, occurring in the Baltic Sea throughout the Bronze Age, although their numbers gradually decline until their final extirpation in the Early Medieval Period c. 1100 CE (Glykou et al. 2021). Two explanations have been proposed for the 1000‐year hiatus. It has been hypothesised that harp seals disappeared completely from the Baltic Sea and later recolonised the region from the White Sea. Alternatively, the harp seals did not disappear, but experienced a substantial reduction in numbers (Glykou et al. 2021). Our mitogenome data revealed little genetic differentiation among Baltic Sea sampling periods, pointing to a genetic continuity from the Mesolithic to the Bronze Age, which contradicts the hypothesis of harp seals disappearing entirely from the Baltic Sea in the Late Neolithic and later recolonising the region in the early Bronze Age. Rather, we speculate whether the hiatus in the zooarchaeological record reflects a substantial decline in the harp seal population, perhaps combined with a reduction of harvesting activities and thus a decline in the deposition of harp seal bones. Human populations in the Baltic Sea region, including areas such as Jutland, the Danish Islands and South Sweden, experienced a decline during the Late Neolithic and increasingly relied on agricultural practices rather than marine resource use (Lewis et al. 2020).

The Bronze Age increase in harp seal zooarchaeological remains following the hiatus likely reflects a population recovery, possibly combined with an influx of immigrants from another (Arctic) harp seal population. Cases of harp seal migrations from the White and Barents Sea southwards along the Norwegian coast to the North Sea, Skagerrak and Kattegat are documented in recent times (Forstén and Alhonen 1975; Hodgetts 1999; Lepiksaar 1964; Nilssen et al. 1998), and similar events could have occurred in the past, providing immigrants to the Baltic harp seal population. These immigrations, combined with the recovery of the local population, may have been fuelled by a period of increased salinity and productivity in the Baltic Sea (Emeis et al. 2003). The increase in harp seal abundance appears to coincide with a simultaneous increase in the abundance of sympatric Baltic grey seals (Fietz et al. 2016), whereas ringed seals appear to have contracted their Baltic range (Ukkonen et al. 2014).

### 4.3 Loss of Genetic Diversity From Mesolithic to Iron Age

The factors causing the Baltic Sea harp seal population to decline during the Late Neolithic and ultimately become extinct in the Early Medieval Period have been much debated, with explanations including changes in sea ice conditions, salinity and productivity; interspecific competition; overexploitation and/or a loss of genetic diversity (Bennike et al. 2008; Glykou et al. 2021; Schmölcke 2008; Storå 2001; Storå and Ericson 2004). We found that the genetic diversity of the Baltic harp seal population decreased from its establishment in the Late Mesolithic to its final extirpation. However, even during the final period of harp seal presence in the Baltic Sea, levels of genetic diversity were comparable to those of contemporary populations, indicating that the loss of diversity was not a causal factor, but rather a consequence of its decline.

Harp seals are gregarious animals, reproducing in large numbers on the pack ice and migrating across vast distances between suitable breeding and feeding sites (Sergeant 1991). In contemporary populations, these migrations likely facilitate some intermixing and the maintenance of genetic diversity. Similarly, while sensitive to environmental change (Johnston et al. 2012), contemporary harp seals may to some extent mitigate the effects by migrating to other regions. For instance, an unusually low extent of sea ice in the breeding seasons in 2010 and 2011 resulted in a large increase in neonatal mortality among Canadian harp seals, but also the movement of animals to regions with more favourable ice conditions (Stenson and Hammill 2014). Moreover, there are several examples of the collapse of regional fish stocks such as capelin leading to mass migrations out of their typical Arctic range in search of food (Nilssen et al. 1998). However, as we have shown, Baltic Sea harp seals were genetically distinct from other populations. As the other populations tracked their habitat northward to the Arctic during the Holocene, Baltic harp seals likely became increasingly isolated and found themselves at a dead end, unable to escape periods of intense harvest and/or adverse environmental conditions.

The zooarchaeological record provides evidence of extensive harvesting by Mesolithic and Neolithic cultures (i.e., Ertebølle, Funnel Beaker and Pitted Ware), which appear to have particularly targeted yearlings (Apel and Storå 2017; Glykou 2014; Glykou et al. 2021; Storå 2001; Storå and Ericson 2004; Ukkonen 2002). Overexploitation of yearlings has also been documented for historic and contemporary harp seal populations in the Arctic, which suffered severe declines until the hunt was regulated and ultimately banned (Hammill et al. 2015). In addition to anthropogenic impacts, periods of low salinity and hence biological productivity would lead to increased intra‐ and interspecific competition, nutritional stress and population declines (Bennike et al. 2008; Schmölcke 2008; Storå 2001; Storå and Ericson 2004). Baltic harp seals experienced substantial reduction in body size during the Neolithic (Glykou et al. 2021; Storå and Ericson 2004), which could point towards density dependence and gradual deterioration of the environment and associated reduction in prey availability. A reduction in body size has also been observed in other seal species reaching carrying capacity or experiencing a decrease in biological productivity (Harding et al. 2018). Similarly, reduction of sea ice as a breeding platform during Holocene warm periods would likely lead to rapid population declines in Baltic harp seals, in particular when occurring over multiple consecutive mating seasons. In this respect, the Medieval Warm Period was probably the final nail in the coffin, leading to the extirpation of an already vulnerable Baltic Sea harp seal population (Glykou et al. 2021).

## Author Contributions

**Maiken Hemme Bro‐Jørgensen:** conceptualization (equal), data curation (lead), formal analysis (equal), investigation (equal), visualization (lead), writing – original draft (equal). **Hans Ahlgren:** formal analysis (equal), investigation (equal), writing – original draft (equal), writing – review and editing (equal). **Aikaterini Glykou:** conceptualization (equal), investigation (supporting), resources (equal), supervision (equal), writing – review and editing (equal). **Emily J. Ruiz‐Puerta:** formal analysis (supporting), writing – review and editing (equal). **Lembi Lõugas:** investigation (supporting), resources (equal), writing – review and editing (supporting). **Anne Birgitte Gotfredsen:** investigation (supporting), resources (equal), writing – review and editing (supporting). **Morten Tange Olsen:** conceptualization (equal), supervision (equal), writing – original draft (equal), writing – review and editing (equal). **Kerstin Lidén:** conceptualization (equal), investigation (supporting), resources (equal), supervision (equal), writing – original draft (equal), writing – review and editing (equal).

## Conflicts of Interest

The authors declare no conflicts of interest.

## Supporting information


Data S1.

## Data Availability

The data that support the findings of this study are openly available in GenBank at https://www.ncbi.nlm.nih.gov/nuccore/PQ827028 reference number [BankIt2909289 ADV1 PQ827028]. https://www.ncbi.nlm.nih.gov/nuccore/PQ827029 reference number [BankIt2909289 ADV9 PQ827029]. https://www.ncbi.nlm.nih.gov/nuccore/PQ827030 reference number [BankIt2909289 AJV52 PQ827030]. https://www.ncbi.nlm.nih.gov/nuccore/PQ827031 reference number [BankIt2909289 ASV55 PQ827031]. https://www.ncbi.nlm.nih.gov/nuccore/PQ827032 reference number [BankIt2909289 ASV56 PQ827032]. https://www.ncbi.nlm.nih.gov/nuccore/PQ827033 reference number [BankIt2909289 ASV58 PQ827033]. https://www.ncbi.nlm.nih.gov/nuccore/PQ827034 reference number [BankIt2909289 BRO49 PQ827034]. https://www.ncbi.nlm.nih.gov/nuccore/PQ827035 reference number [BankIt2909289 BRO51 PQ827035]. https://www.ncbi.nlm.nih.gov/nuccore/PQ827036 reference number [BankIt2909289 BRU44 PQ827036]. https://www.ncbi.nlm.nih.gov/nuccore/PQ827037 reference number [BankIt2909289 BRY1 PQ827037]. https://www.ncbi.nlm.nih.gov/nuccore/PQ827038 reference number [BankIt2909289 BRY3 PQ827038]. https://www.ncbi.nlm.nih.gov/nuccore/PQ827039 reference number [BankIt2909289 BRY5 PQ827039]. https://www.ncbi.nlm.nih.gov/nuccore/PQ827040 reference number [BankIt2909289 GRN4 PQ827040]. https://www.ncbi.nlm.nih.gov/nuccore/PQ827041 reference number [BankIt2909289 HEM1 PQ827041]. https://www.ncbi.nlm.nih.gov/nuccore/PQ827042 reference number [BankIt2909289 HEM2 PQ827042]. https://www.ncbi.nlm.nih.gov/nuccore/PQ827043 reference number [BankIt2909289 HEM3 PQ827043]. https://www.ncbi.nlm.nih.gov/nuccore/PQ827044 reference number [BankIt2909289 HEM4 PQ827044]. https://www.ncbi.nlm.nih.gov/nuccore/PQ827045 reference number [BankIt2909289 HEM5 PQ827045]. https://www.ncbi.nlm.nih.gov/nuccore/PQ827046 reference number [BankIt2909289 HEM6 PQ827046]. https://www.ncbi.nlm.nih.gov/nuccore/PQ827047 reference number [BankIt2909289 HEM15 PQ827047]. https://www.ncbi.nlm.nih.gov/nuccore/PQ827048 reference number [BankIt2909289 JET37 PQ827048]. https://www.ncbi.nlm.nih.gov/nuccore/PQ827049 reference number [BankIt2909289 KAT1 PQ827049]. https://www.ncbi.nlm.nih.gov/nuccore/PQ827050 reference number [BankIt2909289 KAT2 PQ827050]. https://www.ncbi.nlm.nih.gov/nuccore/PQ827051 reference number [BankIt2909289 KOR144 PQ827051]. https://www.ncbi.nlm.nih.gov/nuccore/PQ827052 reference number [BankIt2909289 KOR148 PQ827052]. https://www.ncbi.nlm.nih.gov/nuccore/PQ827053 reference number [BankIt2909289 NEU2 PQ827053]. https://www.ncbi.nlm.nih.gov/nuccore/PQ827054 reference number [BankIt2909289 NEU4 PQ827054]. https://www.ncbi.nlm.nih.gov/nuccore/PQ827055 reference number [BankIt2909289 NEU6 PQ827055]. https://www.ncbi.nlm.nih.gov/nuccore/PQ827056 reference number [BankIt2909289 NEU7 PQ827056]. https://www.ncbi.nlm.nih.gov/nuccore/PQ827057 reference number [BankIt2909289 NEU8 PQ827057]. https://www.ncbi.nlm.nih.gov/nuccore/PQ827058 reference number [BankIt2909289 NEU9 PQ827058]. https://www.ncbi.nlm.nih.gov/nuccore/PQ827059 reference number [BankIt2909289 NEU10 PQ827059]. https://www.ncbi.nlm.nih.gov/nuccore/PQ827060 reference number [BankIt2909289 NEU11 PQ827060]. https://www.ncbi.nlm.nih.gov/nuccore/PQ827061 reference number [BankIt2909289 NEU12 PQ827061]. https://www.ncbi.nlm.nih.gov/nuccore/PQ827062 reference number [BankIt2909289 NEU13 PQ827062]. https://www.ncbi.nlm.nih.gov/nuccore/PQ827063 reference number [BankIt2909289 NEU14 PQ827063]. https://www.ncbi.nlm.nih.gov/nuccore/PQ827064 reference number [BankIt2909289 NEU15 PQ827064]. https://www.ncbi.nlm.nih.gov/nuccore/PQ827065 reference number [BankIt2909289 NEU16 PQ827065]. https://www.ncbi.nlm.nih.gov/nuccore/PQ827066 reference number [BankIt2909289 NEU17 PQ827066]. https://www.ncbi.nlm.nih.gov/nuccore/PQ827067 reference number [BankIt2909289 NEU19 PQ827067]. https://www.ncbi.nlm.nih.gov/nuccore/PQ827068 reference number [BankIt2909289 P127 PQ827068]. https://www.ncbi.nlm.nih.gov/nuccore/PQ827069 reference number [BankIt2909289 P136 PQ827069]. https://www.ncbi.nlm.nih.gov/nuccore/PQ827070 reference number [BankIt2909289 P138 PQ827070]. https://www.ncbi.nlm.nih.gov/nuccore/PQ827071 reference number [BankIt2909289 P139 PQ827071]. https://www.ncbi.nlm.nih.gov/nuccore/PQ827072 reference number [BankIt2909289 P140 PQ827072]. https://www.ncbi.nlm.nih.gov/nuccore/PQ827073 reference number [BankIt2909289 P141 PQ827073]. https://www.ncbi.nlm.nih.gov/nuccore/PQ827074 reference number [BankIt2909289 P143 PQ827074]. https://www.ncbi.nlm.nih.gov/nuccore/PQ827075 reference number [BankIt2909289 P91Q PQ827075]. https://www.ncbi.nlm.nih.gov/nuccore/PQ827076 reference number [BankIt2909289 P95T PQ827076]. https://www.ncbi.nlm.nih.gov/nuccore/PQ827077 reference number [BankIt2909289 SFO31 PQ827077]. https://www.ncbi.nlm.nih.gov/nuccore/PQ827078 reference number [BankIt2909289 SFO32 PQ827078]. https://www.ncbi.nlm.nih.gov/nuccore/PQ827079 reference number [BankIt2909289 SFO33 PQ827079]. https://www.ncbi.nlm.nih.gov/nuccore/PQ827080 reference number [BankIt2909289 SFO34 PQ827080]. https://www.ncbi.nlm.nih.gov/nuccore/PQ827081 reference number [BankIt2909289 SFO35 PQ827081]. https://www.ncbi.nlm.nih.gov/nuccore/PQ827082 reference number [BankIt2909289 SFO35B PQ827082]. https://www.ncbi.nlm.nih.gov/nuccore/PQ827083 reference number [BankIt2909289 SFO38 PQ827083]. https://www.ncbi.nlm.nih.gov/nuccore/PQ827084 reference number [BankIt2909289 SFO39 PQ827084].
