# Supplementary material for: The Evolutionary History of the Extinct Baltic Sea Harp Seal Population
Source: Ecol Evol. 2025 May 8;15(5):e71322. doi: 10.1002/ece3.71322 (PMC12061552; doi:10.1002/ece3.71322)
Supplement: Supplementary file 1 — Data S1. [file ECE3-15-e71322-s001.docx]

**SUPPLEMENTARY MATERIAL**

The evolutionary history of the extinct Baltic Sea harp seal population

Maiken Hemme Bro-Jørgensen^1,2#^, Hans Ahlgren^1#^, Aikaterini Glykou^1^, Emily Johana Ruiz Puerta^2,3^, Lembi Lõugas^4^, Anne Birgitte Gotfredsen^5^, Morten Tange Olsen^2*^, Kerstin Lidén^1*^

**Supplementary Figure 1: Bayesian phylogeny**

**Supplementary Table 1: Sample IDs and metadata**

**Supplementary Text 1: PALEOMIX script**

**Supplementary Text 2: bModelTest settings and results**

**Supplementary Text 3: Beauti and BEAST settings**


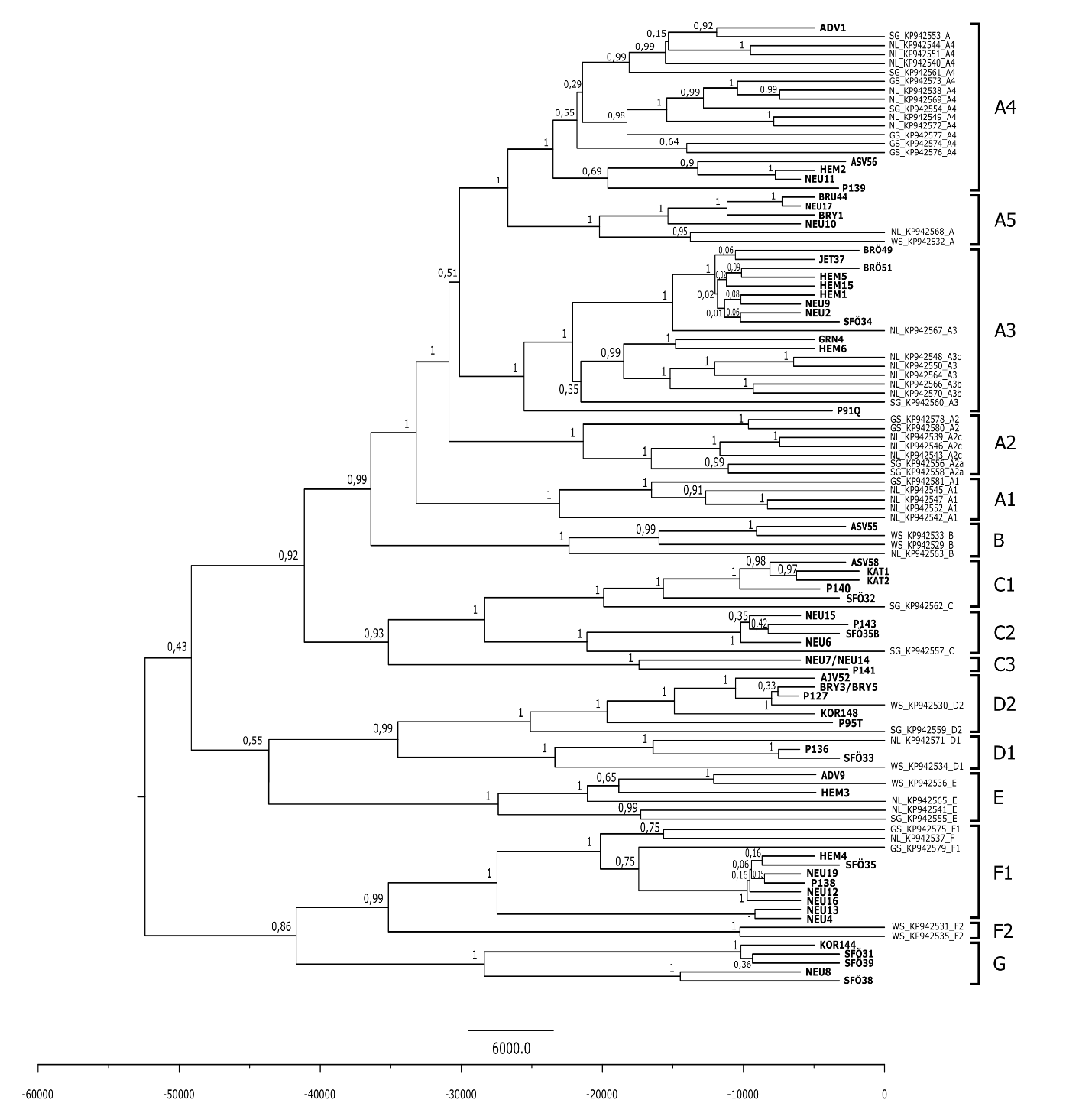


**Supplementary Figure S1**. Bayesian phylogeny of ancient and modern harp seal mitogenomes. Time is in years BP and clades named according the nomenclature proposed by Carr et al. (2015). The first few letters of each sampled denote its geographical origin as per Figure 1, and ancient harp seal samples are highlighted in bold.

**Supplementary Table S1**. Harp seal samples included in the ancient DNA analyses. The dates used in the BEAST analyses reflect an average date based on relative chronology, except when C14 dates are available and dating lab number is provided (Bennike et al., 2008 and Glykou et al. 2020). Sex identification is according to Bro-Jørgensen et al. 2021.

| **Lab ID** | **GenBank accession number** | **Locality** | **Bone element** | **Sex** | **Time period** | **BEAST tip-dates (BP)** | **C14 lab id** |
| --- | --- | --- | --- | --- | --- | --- | --- |
| ADV1 | PQ827028 | Advik, Finnmark, Norway | Scapula | M | Neolithic | 4950 |  |
| ADV9 | PQ827029 | Advik, Finnmark, Norway | Mandibula | M | Neolithic | 4950 |  |
| AJV52 | PQ827030 | Ajvide, Gotland, Sweden | Femur | M | Neolithic | 4801 | Ua-50232 |
| ASV55 | PQ827031 | Asva, Estonia | Humerus | F | Bronze Age | 2660 | Ua-50228 |
| ASV56 | PQ827032 | Asva, Estonia | Humerus | M | Bronze Age | 2750 |  |
| ASV58 | PQ827033 | Asva, Estonia | Humerus | F | Bronze Age | 2670 | Ua-50230 |
| BRÖ49 | PQ827034 | Brömsängsbacken, Åland | Temporal | M | Iron Age | 1130 | Ua-50234 |
| BRÖ51 | PQ827035 | Brömsängsbacken, Åland | Temporal | F | Iron Age | 1053 | Ua-50235 |
| BRU44 | PQ827036 | Brunn, Södermanland, Sweden | Humerus | F | Neolithic | 4950 |  |
| BRY1 | PQ827037 | Bergeby, Finnmark, Norway | Humerus | F | Neolithic | 4950 |  |
| BRY3 | PQ827038 | Bergeby, Finnmark, Norway | Femur | M | Neolithic | 4950 |  |
| BRY5 | PQ827039 | Bergeby, Finnmark, Norway | Femur | M | Neolithic | 4950 |  |
| GRN4 | PQ827040 | Gressbakken, Finnmark, Norway | Femur | M | Neolithic | 4950 |  |
| HEM1 | PQ827041 | Hemmor, Gotland, Sweden | Temporal | F | Neolithic | 4950 |  |
| HEM2 | PQ827042 | Hemmor, Gotland, Sweden | Humerus | ? | Neolithic | 4950 |  |
| HEM3 | PQ827043 | Hemmor, Gotland, Sweden | Temporal | F | Neolithic | 4950 |  |
| HEM4 | PQ827044 | Hemmor, Gotland, Sweden | Temporal | F | Neolithic | 4950 |  |
| HEM5 | PQ827045 | Hemmor, Gotland, Sweden | Temporal | F | Neolithic | 4950 |  |
| HEM6 | PQ827046 | Hemmor, Gotland, Sweden | Temporal | M | Neolithic | 4950 |  |
| HEM15 | PQ827047 | Hemmor, Gotland, Sweden | Temporal | ? | Neolithic | 4825 | Ua-50222 |
| JET37 | PQ827048 | Jettböle, Åland | Temporal | M | Neolithic | 4950 |  |
| KAT1 | PQ827049 | Kattby, Åland | Temporal | M | Iron Age | 1800 |  |
| KAT2 | PQ827050 | Kattby, Åland | Temporal | M | Iron Age | 1800 |  |
| KOR144 | PQ827051 | Korsnäs, Södermanland, Sweden | Bulla tympanica | M | Neolithic | 4950 |  |
| KOR148 | PQ827052 | Korsnäs, Södermanland, Sweden | Bulla tympanica | M | Neolithic | 4950 |  |
| NEU2 | PQ827053 | Neustadt, Ostholstein, Germany | Bulla tympanica | M | Late Mesolithic/ Early Neolithic | 5950 |  |
| NEU4 | PQ827054 | Neustadt, Ostholstein, Germany | Bulla tympanica | M | Late Mesolithic/ Early Neolithic | 5950 |  |
| NEU6 | PQ827055 | Neustadt, Ostholstein, Germany | Bulla tympanica | M | Late Mesolithic/ Early Neolithic | 5950 |  |
| NEU7 | PQ827056 | Neustadt, Ostholstein, Germany | Bulla tympanica | M | Late Mesolithic/ Early Neolithic | 5950 |  |
| NEU8 | PQ827057 | Neustadt, Ostholstein, Germany | Bulla tympanica | M | Late Mesolithic/ Early Neolithic | 5950 |  |
| NEU9 | PQ827058 | Neustadt, Ostholstein, Germany | Bulla tympanica | F | Late Mesolithic/ Early Neolithic | 5950 |  |
| NEU10 | PQ827059 | Neustadt, Ostholstein, Germany | Bulla tympanica | F | Late Mesolithic/ Early Neolithic | 5950 |  |
| NEU11 | PQ827060 | Neustadt, Ostholstein, Germany | Bulla tympanica | F | Late Mesolithic/ Early Neolithic | 5950 |  |
| NEU12 | PQ827061 | Neustadt, Ostholstein, Germany | Bulla tympanica | M | Late Mesolithic/ Early Neolithic | 5950 |  |
| NEU13 | PQ827062 | Neustadt, Ostholstein, Germany | Bulla tympanica | F | Late Mesolithic/ Early Neolithic | 5950 |  |
| NEU14 | PQ827063 | Neustadt, Ostholstein, Germany | Bulla tympanica | F | Late Mesolithic/ Early Neolithic | 5950 |  |
| NEU15 | PQ827064 | Neustadt, Ostholstein, Germany | Bulla tympanica | F | Late Mesolithic/ Early Neolithic | 5950 |  |
| NEU16 | PQ827065 | Neustadt, Ostholstein, Germany | Bulla tympanica | F | Late Mesolithic/ Early Neolithic | 5950 |  |
| NEU17 | PQ827066 | Neustadt, Ostholstein, Germany | Bulla tympanica | M | Late Mesolithic/ Early Neolithic | 5950 |  |
| NEU19 | PQ827067 | Neustadt, Ostholstein, Germany | Bulla tympanica | F | Late Mesolithic/ Early Neolithic | 5950 |  |
| P127 | PQ827068 | Flynderhage, Denmark | Pars petrosa | M | Late Mesolithic/ Early Neolithic | 6224 | LuS-6133 |
| P136 | PQ827069 | Ronæs Skov, Denmark | Maxilla | M | Late Mesolithic/ Early Neolithic | 6188 | LuS-6136 |
| P138 | PQ827070 | Gudumlund, Denmark | Pars petrosa | F | Early Neolithic | 5876 | LuS-6130 |
| P139 | PQ827071 | Argusgrunden, Denmark | Occipital condyle | F | Bronze Age | 3455 | LuS-6122 |
| P140 | PQ827072 | Krabbesholm, Denmark | Pars petrosa | F | Late Neolithic | 4859 | LuS-6138 |
| P141 | PQ827073 | Aabenraa, Denmark | Pars petrosa | M | Late Bronze Age/ Early Iron Age | 2881 | LuS-6118 |
| P143 | PQ827074 | Aabenraa Fjord, Denmark | Pars petrosa | M | Late Bronze Age/ Early Iron Age | 2808 | LuS-6117 |
| P91Q | PQ827075 | Qeqertasussuk, Greenland | Bulla tympanica | F | Saqqaq culture | 3700 |  |
| P95T | PQ827076 | Qeqertasussuk, Greenland | Bulla tympanica | M | Saqqaq culture | 3700 |  |
| SFÖ31 | PQ827077 | Stora Förvar, Gotland, Sweden | Femur | M | Bronze Age | 3006 | Ua-50224 |
| SFÖ32 | PQ827078 | Stora Förvar, Gotland, Sweden | Femur | M | Bronze Age | 3200 |  |
| SFÖ33 | PQ827079 | Stora Förvar, Gotland, Sweden | Femur | M | Bronze Age | 3200 |  |
| SFÖ34 | PQ827080 | Stora Förvar, Gotland, Sweden | Femur | M | Bronze Age | 3200 |  |
| SFÖ35 | PQ827081 | Stora Förvar, Gotland, Sweden | Femur | M | Bronze Age | 3200 |  |
| SFÖ35B | PQ827082 | Stora Förvar, Gotland, Sweden | Bulla tympanica | F | Bronze Age | 3200 |  |
| SFÖ38 | PQ827083 | Stora Förvar, Gotland, Sweden | Femur mid | F | Bronze Age | 3200 |  |
| SFÖ39 | PQ827084 | Stora Förvar, Gotland, Sweden | Humerus mid | F | Bronze Age | 3388 | Ua-50226 |

**Supplementary Text 1: Palaeomix script**

# -*- mode: Yaml; -*-

# Timestamp: 2018-05-02T10:31:49.094715

#

# Default options.

# Can also be specific for a set of samples, libraries, and lanes,

# by including the "Options" hierarchy at the same level as those

# samples, libraries, or lanes below. This does not include

# "Features", which may only be specific globally.

Options:

# Sequencing platform, see SAM/BAM reference for valid values

Platform: Illumina

# Quality offset for Phred scores, either 33 (Sanger/Illumina 1.8+)

# or 64 (Illumina 1.3+ / 1.5+). For Bowtie2 it is also possible to

# specify 'Solexa', to handle reads on the Solexa scale. This is

# used during adapter-trimming and sequence alignment

QualityOffset: 33

# Split a lane into multiple entries, one for each (pair of) file(s)

# found using the search-string specified for a given lane. Each

# lane is named by adding a number to the end of the given barcode.

SplitLanesByFilenames: yes

# Compression format for FASTQ reads; 'gz' for GZip, 'bz2' for BZip2

CompressionFormat: gz

# Settings for trimming of reads, see AdapterRemoval man-page

AdapterRemoval:

# Adapter sequences, set and uncomment to override defaults

# --adapter1: AGATCGGAAGAGCACACGTCTGAACTCCAGTCACNNNNNNATCTCGTATGCCGTCTTCTGCTTG

# --adapter2: AGATCGGAAGAGCGTCGTGTAGGGAAAGAGTGTAGATCTCGGTGGTCGCCGTATCATT

# Some BAM pipeline defaults differ from AR defaults;

# To override, change these value(s):

--mm: 3

--minlength: 25

# Extra features enabled by default; change 'yes' to 'no' to disable

--collapse: yes

--trimns: yes

--trimqualities: yes

# Settings for aligners supported by the pipeline

Aligners:

# Choice of aligner software to use, either "BWA" or "Bowtie2"

Program: BWA

# Settings for mappings performed using BWA

BWA:

# One of "backtrack", "bwasw", or "mem"; see the BWA documentation

# for a description of each algorithm (defaults to 'backtrack')

Algorithm: backtrack

# Filter aligned reads with a mapping quality (Phred) below this value

MinQuality: 30

# Filter reads that did not map to the reference sequence

FilterUnmappedReads: no

# May be disabled ("no") for aDNA alignments with the 'aln' algorithm.

# Post-mortem damage localizes to the seed region, which BWA expects to

# have few errors (sets "-l"). See http://pmid.us/22574660

UseSeed: no

# Additional command-line options may be specified for the "aln"

# call(s), as described below for Bowtie2 below.

# Settings for mappings performed using Bowtie2

Bowtie2:

# Filter aligned reads with a mapping quality (Phred) below this value

MinQuality: 0

# Filter reads that did not map to the reference sequence

FilterUnmappedReads: yes

# Examples of how to add additional command-line options

# --trim5: 5

# --trim3: 5

# Note that the colon is required, even if no value is specified

--very-sensitive:

# Example of how to specify multiple values for an option

# --rg:

# - CN:SequencingCenterNameHere

# - DS:DescriptionOfReadGroup

# Mark / filter PCR duplicates. If set to 'filter', PCR duplicates are

# removed from the output files; if set to 'mark', PCR duplicates are

# flagged with bit 0x400, and not removed from the output files; if set to

# 'no', the reads are assumed to not have been amplified. Collapsed reads

# are filtered using the command 'paleomix rmdup_duplicates', while "normal"

# reads are filtered using Picard MarkDuplicates.

PCRDuplicates: filter

# Command-line options for mapDamage; note that the long-form

# options are expected; --length, not -l, etc. Uncomment the

# "mapDamage" line adding command-line options below.

mapDamage:

# By default, the pipeline will downsample the input to 100k hits

# when running mapDamage; remove to use all hits

--downsample: 100000

# Set to 'yes' exclude a type of trimmed reads from alignment / analysis;

# possible read-types reflect the output of AdapterRemoval

ExcludeReads:

# Exclude single-end reads (yes / no)?

Single: no

# Exclude non-collapsed paired-end reads (yes / no)?

Paired: no

# Exclude paired-end reads for which the mate was discarded (yes / no)?

Singleton: no

# Exclude overlapping paired-ended reads collapsed into a single sequence

# by AdapterRemoval (yes / no)?

Collapsed: no

# Like 'Collapsed', but only for collapsed reads truncated due to the

# presence of ambiguous or low quality bases at read termini (yes / no).

CollapsedTruncated: no

# Optional steps to perform during processing.

Features:

# Generate BAM without realignment around indels (yes / no)

RawBAM: no

# Generate indel-realigned BAM using the GATK Indel realigner (yes / no)

RealignedBAM: yes

# To disable mapDamage, write 'no'; to generate basic mapDamage plots,

# write 'plot'; to build post-mortem damage models, write 'model',

# and to produce rescaled BAMs, write 'rescale'. The 'model' option

# includes the 'plot' output, and the 'rescale' option includes both

# 'plot' and 'model' results. All analyses are carried out per library.

mapDamage: model

# Generate coverage information for the raw BAM (wo/ indel realignment).

# If one or more 'RegionsOfInterest' have been specified for a prefix,

# additional coverage files are generated for each alignment (yes / no)

Coverage: yes

# Generate histogram of number of sites with a given read-depth, from 0

# to 200. If one or more 'RegionsOfInterest' have been specified for a

# prefix, additional histograms are generated for each alignment (yes / no)

Depths: yes

# Generate summary table for each target (yes / no)

Summary: yes

# Generate histogram of PCR duplicates, for use with PreSeq (yes / no)

DuplicateHist: yes

# Map of prefixes by name, each having a Path key, which specifies the

# location of the BWA/Bowtie2 index, and optional label, and an option

# set of regions for which additional statistics are produced.

Prefixes:

# Replace 'NAME_OF_PREFIX' with name of the prefix; this name

# is used in summary statistics and as part of output filenames.

'NAME_OF_PREFIX':

# Replace 'PATH_TO_PREFIX' with the path to .fasta file containing the

# references against which reads are to be mapped. Using the same name

# as filename is strongly recommended (e.g. /path/to/Human_g1k_v37.fasta

# should be named 'Human_g1k_v37').

Path: /'PATH_TO_PREFIX'.fasta

# (Optional) Uncomment and replace 'PATH_TO_BEDFILE' with the path to a

# .bed file listing extra regions for which coverage / depth statistics

# should be calculated; if no names are specified for the BED records,

# results are named after the chromosome / contig. Change 'NAME' to the

# name to be used in summary statistics and output filenames.

RegionsOfInterest:

chrMT: /'PATH_TO_BEDFILE'.bed

# Mapping targets are specified using the following structure. Uncomment and

# replace 'NAME_OF_TARGET' with the desired prefix for filenames.

#NAME_OF_TARGET:

# Uncomment and replace 'NAME_OF_SAMPLE' with the name of this sample.

# NAME_OF_SAMPLE:

# Uncomment and replace 'NAME_OF_LIBRARY' with the name of this sample.

# NAME_OF_LIBRARY:

# Uncomment and replace 'NAME_OF_LANE' with the name of this lane,

# and replace 'PATH_WITH_WILDCARDS' with the path to the FASTQ files

# to be trimmed and mapped for this lane (may include wildcards).

# NAME_OF_LANE: PATH_WITH_WILDCARDS

**Supplementary Text 2: bModelTest settings and results**

1# Partitions: Insert data. No partitions for this case.

2# Tip Dates: add tip dates for each sample using .txt file prepared for Beauti files.

3# Site model: select BEAST Model Test and check box for estimate. Leave the other options as default.

4# Clock model: Set as Relaxed Clock Exponential with a clock. Rate 2.9E-4

5#Priors:

> Tree.t: Coalescent Exponential Population.

>Leave the other priors options as in default. See Supplementary Figure 2.

5# MCMC:

>300 million iterations

>Store every: default

>Pre Burnin of 10%

> Num Initialization Attemps: default

> tracelog, screenlog and treelog were set as default (1000)

For more information, please see the following figures of settings and results. a)**
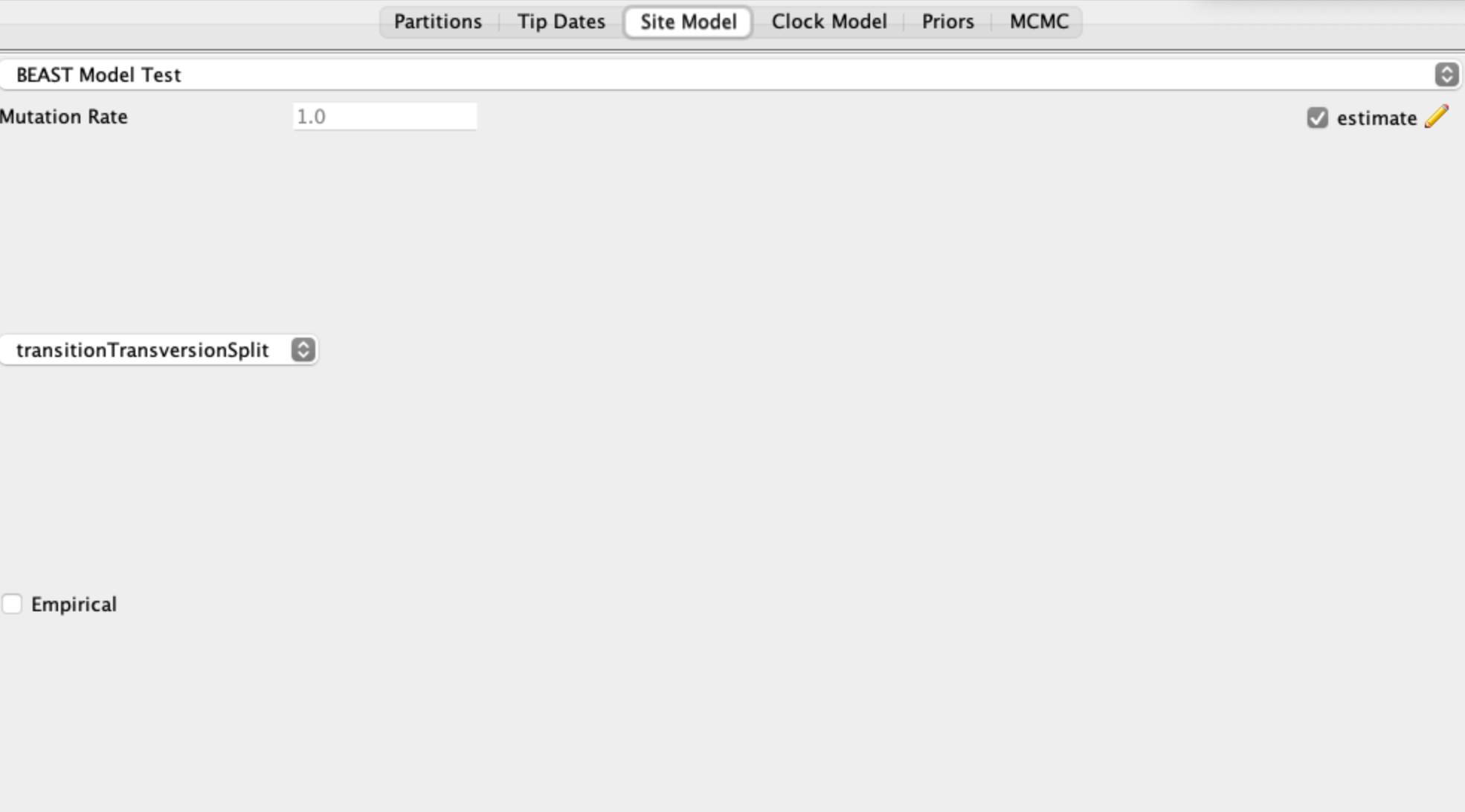
**b) **
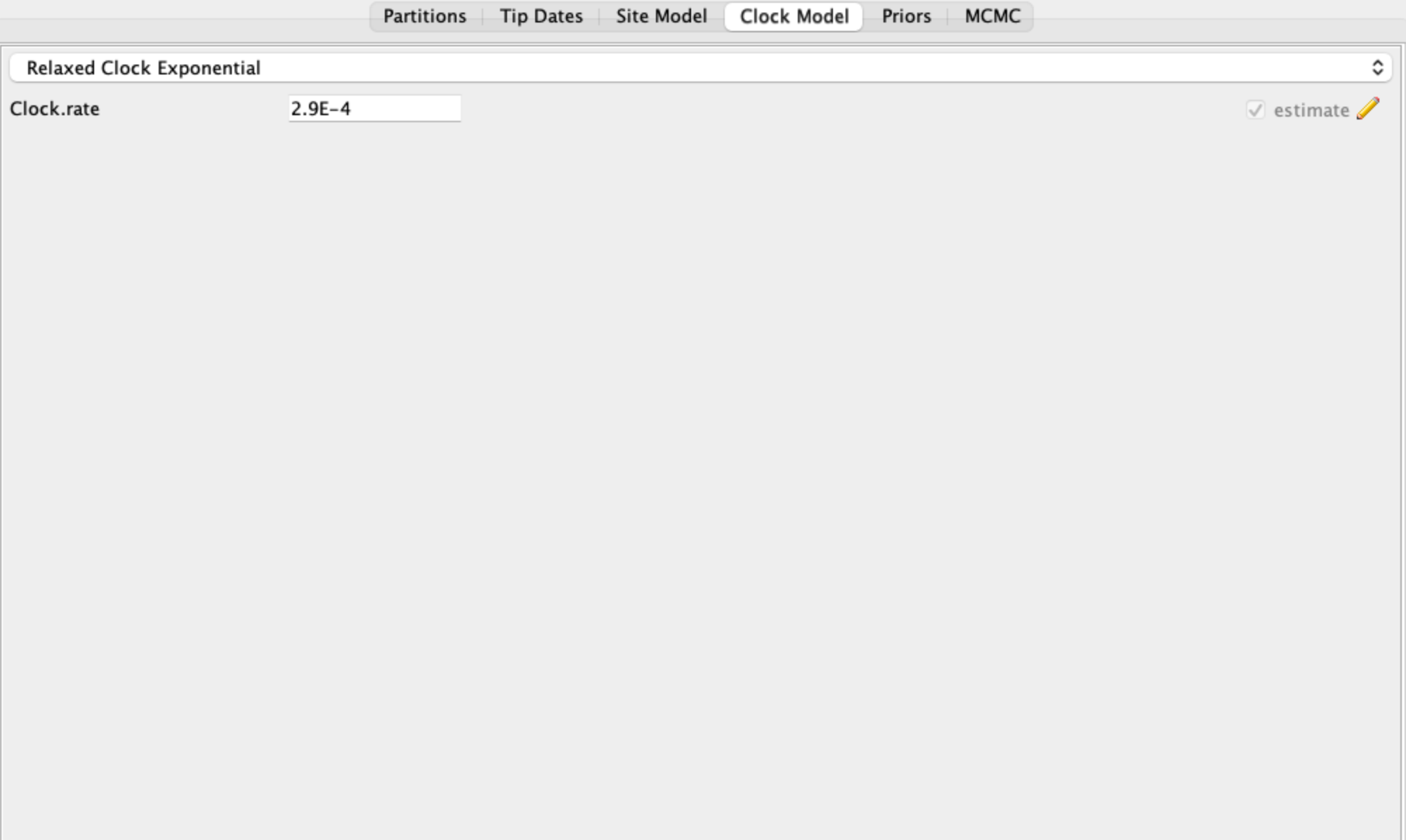
** c)
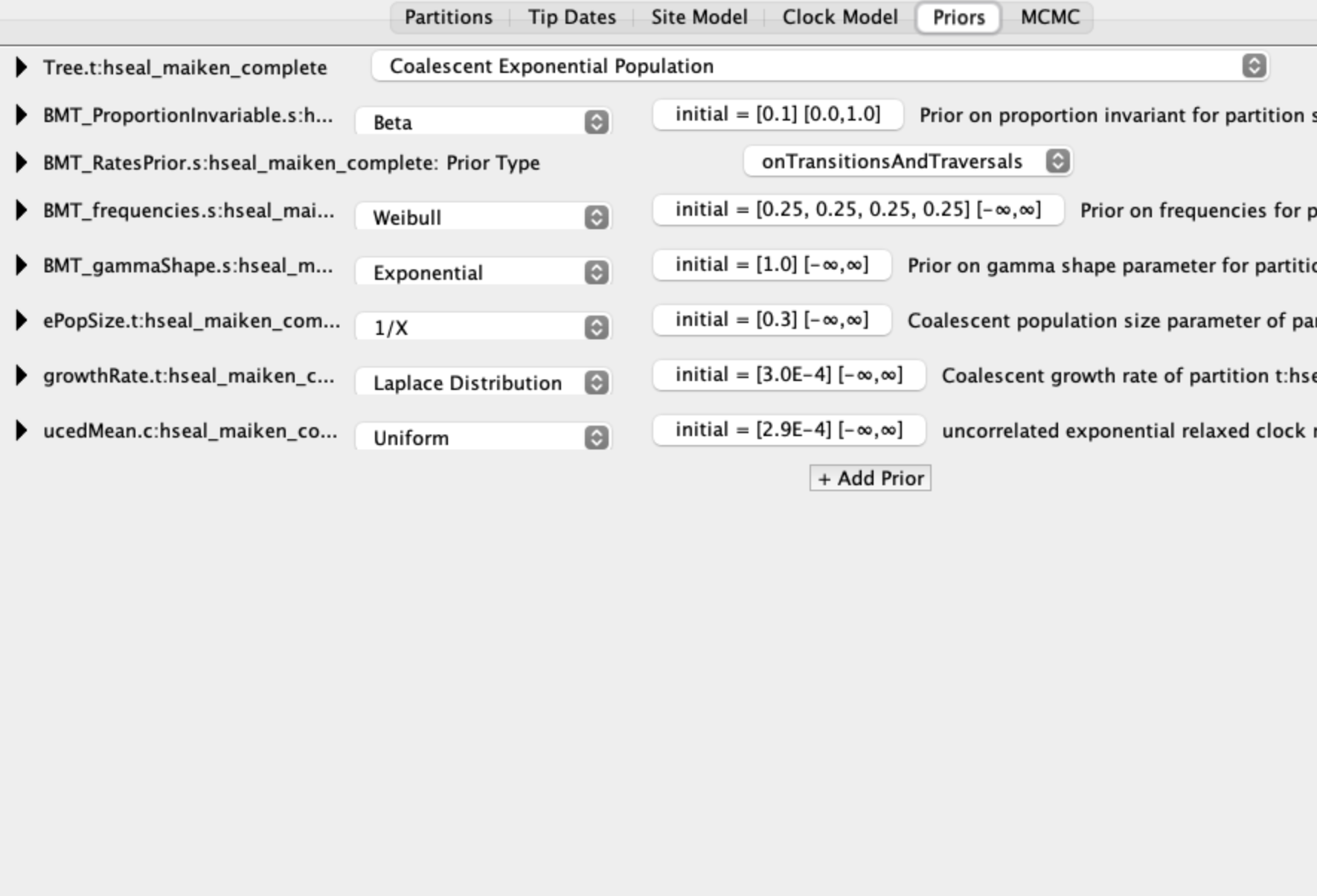
d)
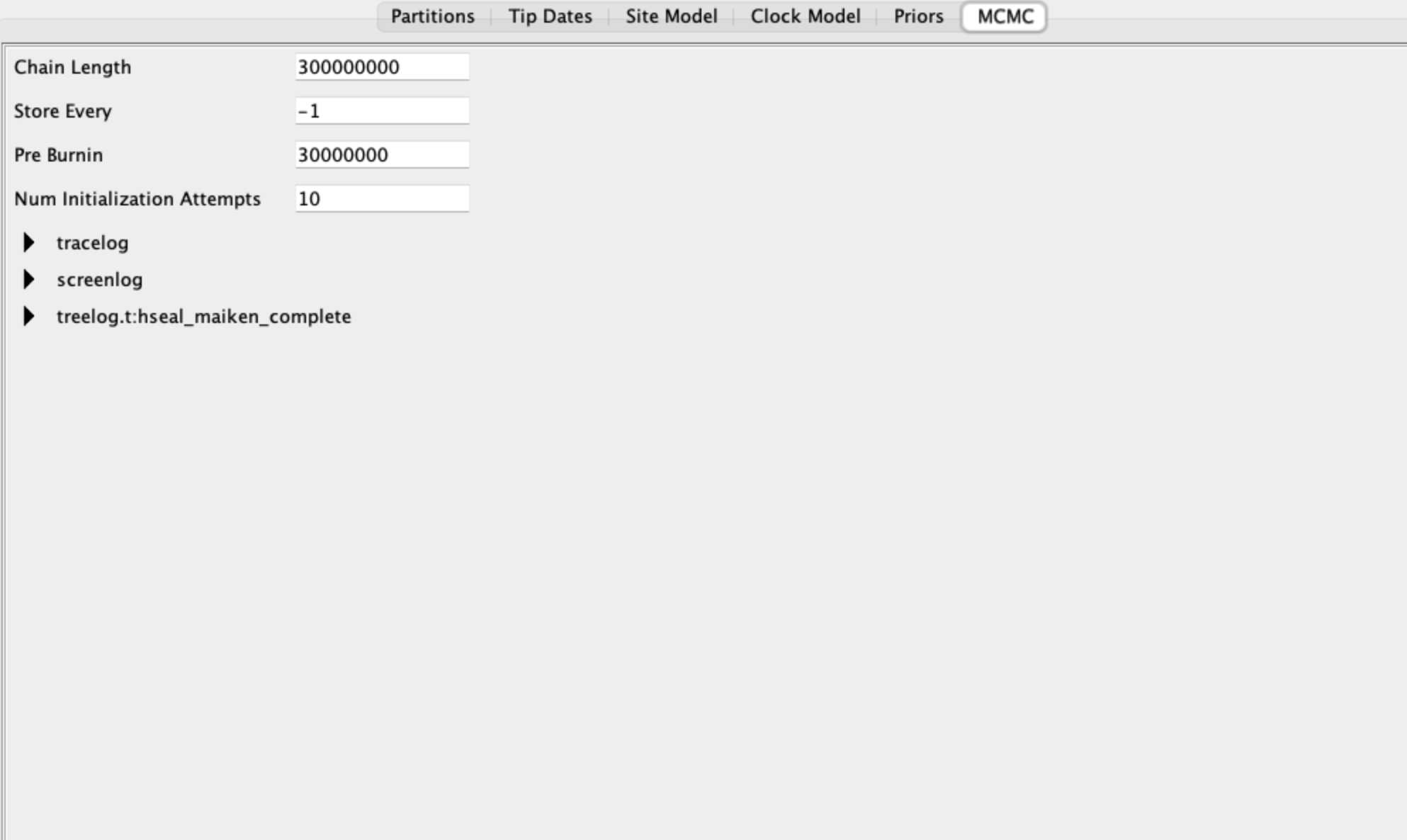

**Supplementary Text 3: BEAST settings**

Settings for BEAST analysis are same as described in Supplementary Text 2 and Supplementary Figure 2. The implementation of BModeltest output was applied in the site model settings.

1# Site Model:

>Set up as Gamma Site Model

>Leave as default:Substitution rate, Gamma Category Count, and Proportion Invariant

> Subst Model: select GTR

> Rate AC: set to 1

> Rate AG: set to 2

> Rate AT: set to 3

> Rate CG: set to 1

> Rate CT: set to 2

> Rate GT: set to 1

> Frequencies: set as default, Estimated
